# Human brucellosis: sero-prevalence and associated risk factors in agro-pastoral communities of Kiboga District, Central Uganda

**DOI:** 10.1186/s12889-015-2242-z

**Published:** 2015-09-15

**Authors:** Gabriel Tumwine, Enock Matovu, John David Kabasa, David Okello Owiny, Samuel Majalija

**Affiliations:** Department of Biomolecular Resources and Biolab Sciences, College of Veterinary Medicine, Animal Resources and Biosecurity, Makerere University, P.O. Box 7062, Kampala, Uganda; Department of Biosecurity, Ecosystem and Veterinary Public Health, College of Veterinary Medicine, Animal Resources and Biosecurity, Makerere University, Kampala, Uganda; Department of Biotechnical and Diagnostic Sciences, College of Veterinary Medicine, Animal Resources and Biosecurity, Makerere University, Kampala, Uganda

## Abstract

**Background:**

Brucellosis remains a neglected zoonotic disease among agro-pastoral communities where unprocessed milk and milk products are consumed. A cross-sectional study was carried out in Kiboga district to determine the seroprevalence and risk factors associated with human brucellosis in communities where livestock rearing in a common practice.

**Methods:**

A total of 235 participants were involved in the study. Blood samples from the participants were collected and screened for *Brucella* using Serum Agglutination Test and Rose Bengal Plate Test. A questionnaire was used to collect data on socio-demographic characteristics and human brucellosis related risk factors.

**Results:**

Human *Brucella* seroprevalence was at 17.0 % (*n* = 235). The prevalence was highest among males (20.5 %, *n* = 78) and the elderly - above 60 years (22.2 %, *n* = 18). Residence in rural areas (OR 3.16, 95 % CI: 1.16–8.56), consuming locally processed milk products (OR 2.54, 95 % CI: 1.12–5.78) and being single (OR 2.44, 95 % CI: 1.05–5.68), were associated with increased risk of brucellosis.

**Discussion:**

Human brucellosis seroprevalence was high at 17 %, this was parallel with animal brucellosis prevalence that has been reported to range from 10.2 % to 25.7 % in cattle in the region. The participants were from communities known to habitually consume raw milk and milk products, know to process milk products using bare hands which are major risk factors for brucellosis in humans. This also explains why consumption of unpasteurized milk products was associated with the occurrence of brucellosis in study area. This strengthened the argument that humans get infected through consumption of contaminated animal products as reported in other earlier studies. Males and elderly being more affected because of traditional roles of these groups they play in livestock care and management. The single were also to be more associated to brucellosis, due to the fact that this group consume milk and milk products more as it is readily available in the informal markets as highly nutritious fast foods in this community as also observed in USA.

**Conclusions:**

Brucellosis is highly prevalent in Kiboga district, and therefore, an important public health problem. The transmission risk was aggravated by consumption of unpasteurized milk products, residing in rural settings and being single. There is a need to initiate screening, treat infected humans early, and educate the public about risk factors and appropriate preventive measures of brucellosis.

## Background

Brucellosis is one of the important neglected bacterial zoonotic diseases that has affected animals and humans for decades [1, 2]. The disease is caused by bacterial agent of genus *Brucella*. Human brucellosis is caused mainly by *Brucella abortus*, *B. melitensis* and *B. suis,* also the main causes of brucellosis in cattle, goats/sheep and pigs respectively [1, 3]. Wild life animals are also equally affected and these may act as reservoirs to both domestic animals and humans [4]. Human infection is through contact with infected animals and ingestion of contaminated animal products such as milk, meat, or carcasses [5–7]. Brucellosis therefore, remains an occupational hazard to veterinarians, slaughter house workers, farmers and laboratory personnel, who commonly get in contact with the animals [8]. However, few cases of human to human transmission have been reported [9].

Human brucellosis has a wide clinical spectrum, presenting various diagnostic difficulties because it mimics many other diseases for example malaria, typhoid, rheumatic fever, joint diseases and other conditions causing pyrexia [10–14]. The disease manifests with continued, intermittent or irregular fever (hence the name undulant fever), headache, weakness, profuse sweating, chills, arthralgia, depression, weight loss, hepatomegaly, and splenomegaly and generalized aching. Cases of arthritis, spondylitis, osteomyelitis, epididymitis, orchitis, and in severe cases neurobrucellosis, liver abscesses, and endocarditis have also been reported in humans [15, 16]. Endocarditis and infection of the aortic valves and other multiple valves with *Brucella* has been reported to cause an average of 5 % case mortality rate in humans [17, 18]. Chronic cases are common and are due to the *Brucella* pathogen’s ability to survive and multiply in macrophages, the major cells of immune response [19]. In humans, under diagnosis and under reporting is the major cause and consequence of chronic debilitating cases of the disease [20].

Globally, over 500,000 human cases of brucellosis per year are reported [21]. In sub-Saharan Africa, brucellosis prevalence is unclear and poorly understood with varying reports from country to country, geographical regions as well as animal factors [11]. Less has been reported in humans than in animals. In cattle for instance, a prevalence ranging from 10.2 to 25.7 % is reported across sub-Saharan Africa [22]. In Tanzania, human brucellosis prevalence has been recorded to range from 1 to 5 % [23]. However, the prevalence reported in Togo (41 %) was outside this range [24]. High prevalence of over 40 % has also been reported in Libya [25]. In Uganda, animal sero-prevalence of 4 to 26 % has been reported from 3 districts in east, central and western Uganda [26–28]. A seroprevalence of 13.3 % in urban settings of Uganda, has been reported among patients who reported with febrile illnesses [12] and a seroprevalence of 7 and 12 % among the butchers in Mbarara and Kampala districts respectively [29].

The humans’ exposure to infected animals and animal products increases the risk of acquiring brucellosis [5, 6, 30] as seen in cases of butchers [29]. Therefore, people in pastoral communities are at high risk of infection due to constant contact with animals and their products.

In Uganda, over 80 % of people rely on agriculture with larger proportion depending on animals. In pastoral areas, people almost entirely depend on livestock for their livelihood [11]. These animals pose a public health threat to humans since cases of animal brucellosis in Uganda have been reported to be high [27, 28].

In Uganda, most (92 %) of the unpasteurized milk is marketed and sold in informal markets [31]. Thus, cases of humans’ brucellosis are expected to be high. The disease tends to go unnoticed since many patients and clinicians treat it as malaria, typhoid, joint diseases and other conditions that cause pyrexia [11, 12, 32]. These diagnostic challenges coupled with poor health infrastructures in agro-pastoral communities in Uganda lead to chronic, debilitating and common cases of brucellosis [11, 20, 21]. Control measures remain a challenge in resource poor countries [33]. In Uganda, few studies have been carried out to establish prevalence of human brucellosis. Therefore, the aim of this study was to establish the prevalence and associated risk factors for brucellosis among humans in Kiboga district, one of the agro-pastoral districts in Uganda. Baseline information necessary in designing prevention and control strategies against human brucellosis in Uganda was obtained.

## Methods

### Study area

The study was carried out in Kiboga district in central Uganda. The study area is located in the cattle corridor of Uganda. In this region, cattle and goat rearing is a common practice with small herds/flocks, ranging from 10–50 animal per household with average of 19 cattle as well as large herds/flocks over 50 animals kept under communal grazing and paddocking systems [34]. The population is mainly served by Kiboga main hospital, Bukomero Health Centre IV and other lower health centers. This study involved patients who visited the two main health facilities.

### Ethical considerations

This study was peer reviewed and approved by both the Uganda National Council of Science and Technology (UNCST). The approval number is HS 165. In addition, it was approved by the Institutional Review Board (IRB) from the College of Health Sciences (CHS), Makerere University (REC Ref N0. 2011–195). The blood samples were obtained for the study after obtaining an informed verbal and written consent from participants and legal guardians in presence of independent witnesses. Each participant was assigned a unique code to maintain anonymity of the samples. Participants that tested positive for *Brucella*, were treated based on National guidelines implemented at the health facility.

### Study design, participant involvement, sample collection and laboratory tests

A cross-sectional survey was conducted among 235 people who visited the two hospitals from June to July 2014 and who had stayed in Kiboga district for the past three months. Both males and females who had come to hospital for some other ailment as well as those apparently healthy were involved in the study after receiving informed consent. The sample size was obtained based on Martin et al. [35] at 95 % confidence interval (CI) and presumed sero-prevalence of 12 % [29].

An open- and closed-ended questionnaire was developed and administered to collect information about factors hypothesized to influence the spread and persistence of brucellosis in humans. The questionnaire included socio-demographic factors (sex, age, marital status, religion, education level, and occupation), knowledge of brucellosis, contact with animals and animal products, participant’s involvement in milking, sharing water sources with animals, assisting animals to give birth or drink animal urine.

With the help of trained medical personnel, a blood sample (5–7 ml) was obtained from each participant following venipucture with the disposable needles and vacutainers. The blood samples were clearly labeled with a specific code, date of collection and location. The specimens were kept at room temperature for 30 min, then at 4 °C for 24 h. Later, they were transported on ice to the central diagnostic laboratory at the College of Veterinary Medicine, Animal Resources and Biosecurity, Makerere University for further processing.

In the laboratory, serum samples were extracted and screened for anti-*Brucella* antibodies using commercial kits of the standard Rose Bengal Plate Test (RBPT) and later confirmed using Serum Agglutination Test (SAT) [36]. *Brucella abortus* antigens and their positive and negative control sera (Veterinary Laboratory Agency, VLA, Weybridge, United Kingdom) were used to detect the *Brucella* antibodies following the manufactures’ instructions. Only samples that gave signals for both RBPT and SAT were considered positive since no single test is appropriate in all epidemiological situations due to problems of sensitivity and or specificity of the tests as recommended by OIE and other reports [37–39].

### Data management and analysis

The data obtained were coded and entered in the Microsoft Excel 2010, exported, cleaned and analysed using STATA version 12 (Stata Corp., College Station, TX, USA). Descriptive analysis was used to summarize the data - in form of frequencies and percentages. Bivariate data analysis was conducted to establish the risk factors associated with brucellosis in humans and odds ratios were obtained at 95 % confidence intervals (CI). Independent variables which had *p*-values <0.2 at bivariate analysis and were previously reported to be associated with brucellosis in humans were considered for inclusion in multivariate analysis. Eligible variables were assessed for collinearity. A multivariable logistic regression was performed to establish associations between risk factors and brucellosis occurrence in humans. We also tested for interaction terms.. The Hosmer and Lemeshow goodness-of-fit test was used to assess the model fit.

## Results

### Socio-demographic characteristics

Table 1 presents descriptive characteristics of the participants. A total of 235 human participants were involved in the study. The participants’ age ranged from 7 to 78 years with overall average age of 32.7 (±14.7) years with the majority 74.5 % (*n* = 175) in 20–59 age group. Most of the participants were female 66.8 % (*n* = 157), while 67.7 % (*n* = 159) were from rural areas of Kiboga. Farmers comprised 64.3 % (*n* = 151) and 59.6 % (*n* = 114) kept animals (cattle, goats and sheep) in their homes. The majority of the participants 75.3 % (*n* = 177) had formal education; 66.0 % (*n* = 155) were Christians and almost half 49.8 % (*n* = 117) were married (Table 1). Less than half 41.7 %, (*n* = 98) of the participants were knowledgeable about the disease.

**Table 1:** Descriptive socio-demographic characteristics and brucella sero-prevalence of among participants

Characteristics	% participants (*N* = 235)	Frequency (n)	+ve Brucella (n, %)	*P*-value
Sex				0.32
Male	33.2	78.0	16 (20.5)	
Female	66.8	157.0	24 (15.3)	
Age group (years)				0.95
Below 13	5.1	12.0	2 (16.7)	
13–19	12.8	30.0	5 (16.7)	
20–59	74.5	175.0	29 (16.6)	
Above 60	7.7	18.0	4 (22.2)	
Residence				0.01
Urban	32.3	76.0	6 (7.9)	
Rural	67.7	159.0	34 (21.4)	
Occupation				0.25
Farmer	64.3	151.0	31 (20.5)	
Unemployed^a^	16.2	38.0	5 (13.2)	
Employed^b^	7.7	18.0	2 (11.1)	
Self-employed^c^	11.9	28.0	2 (7.1)	
Education				0.62
None	24.7	58.0	9 (15.5)	
Primary	48.1	113.0	22 (19.5)	
Secondary and above	27.2	64.0	9 (14.1)	
Marital status				0.15
Married	49.8	117.0	17 (14.5)	
Single	30.6	72.0	16 (22.2)	
Widowed	7.7	18.0	5 (27.8)	
Divorced	11.9	28.0	2 (7.1)	
Religion				0.87
Muslim	15.3	36.0	7 (19.4)	
Christian	66.0	155.0	25 (16.1)	
Others	18.7	44.0	8 (18.2)	
Animals at home				0.44
No	40.4	95.0	14 (14.7)	
Yes	59.6	140.0	26 (18.6)	
Knowledge of brucellosis				0.81
No	58.3	137	24 (17.5)	
Yes	41.7	98	16 (16.3)	

^a^House wives, Students and Others

^b^Civil servants and professionals

^c^Boda boda riders, Carpenters, small businesses

### Prevalence of brucellosis

Table 1 also shows that the overall sero-prevalence of brucellosis among the 235 participants was 17 % (*n* = 40). The sero-prevalence varied among males (20.5 %, *n* = 78) and females (15.3 %, *n* = 157) and was higher in participants above 60 year (22.2 %, *n* = 18). The prevalence was also higher (21.4 %, *n* = 159) in rural than urban (7.9 %, *n* = 76) dwellers. The prevalence of brucellosis of 18.6 % (*n* = 140), 19.4 % (*n* = 36), 20.5 % (*n* = 151), and 27.8 % (*n* = 18), were respectively recorded among participants rearing animals in their homes, Muslims, farmers, and the widowed. Of 41.7 % that were aware of the disease, 16.3 % (*n* = 98) tested positive for *Brucella*.

Most of the social-demographic factors were not associated (*p* > 0.05) with the occurrence of brucellosis among humans except the place of residence. Those from rural areas were associated (*p* = 0.01) with *Brucella* positive cases than their urban counterparts.

### Risk factors for occurrence of brucellosis in humans

Table 2 presents the potential risk factors that may be attributed to the occurrence of brucellosis among humans. Overall, the participants who reported to have been exposed to the potential risk factors, had more cases of brucella positive. These included; consuming of raw milk (20 %, *n* = 30), close contact with animals at home (20.5 %, *n* = 122), milking animals (21.1 %, *n* = 38), exposure to animals at work (21.4 %, *n* = 56), handling of aborted fetuses (21.4 %, *n* = 14), consuming milk products (22.4 %, *n* = 125), processing local milk products (25.8 %, *n* = 62), assisting animals giving birth (29.2 %, *n* = 24), slaughtering of animals at home (29.4 %, *n* = 17), sharing water points with animals (33.3 %, *n* = 15) and drinking animal’s urine (42.9 %, *n* = 7).

**Table 2:** Potential risk factors assessed by bivariate analysis for brucellosis seropositivity in humans (*n* = 235)

Variable		% participants (*n* = 235)	Frequency	+ve Brucella (n, %)	Unadjusted OR (95 % CI)	*P*-value
Animal exposure at home	No	48.1	113	15 (13.3)		
	Yes	51.9	122	25 (20.5)	1.68 (0.84, 3.39)	0.14
Animal exposure at work	No	76.2	179	28 (15.6)		
	Yes	23.8	56	12 (21.4)	1.47 (0.69, 3.13)	0.32
Handling of aborted fetuses	No	94.0	221	37 (16.7)		
	Yes	6.0	14	3 (21.4)	1.36 (0.36, 5.10)	0.65
Slaughter animals	No	92.8	218	35 (16.1)		
	Yes	7.2	17	5 (29.4)	2.18 (0.72, 6.57)	0.17
Milking	No	83.8	197	32 (16.2)		
	Yes	16.2	38	8 (21.1)	1.38 (0.58, 3.27)	0.47
Processing Milk products	No	73.6	173	24 (13.9)		
	Yes	26.4	62	16 (25.8)	2.16 (1.06, 4.41)	0.035
Consuming raw milk	No	87.2	205	34 (16.6)		
	Yes	12.8	30	6 (20.0)	1.26 (0.48, 3.31)	0.64
Consuming milk products	No	46.8	110	12 (10.9)		
	Yes	53.2	125	28 (22.4)	2.36 (1.13, 4.90)	0.022
Assist animals giving births	No	89.8	211	33 (15.6)		
	Yes	10.2	24	7 (29.2)	2.22 (0.85, 5.77)	0.102
Drinking animal’s urine	No	97.0	228	37 (16.2)		
	Yes	3.0	7	3 (42.9)	3.87 (0.83, 18.02)	0.084
Sharing water points with animals	No	93.6	220	35 (15.9)		
	Yes	6.4	15	5 (33.3)	2.64 (0.85, 8.20)	0.093

At bivariate analysis, the processing of local milk products *e.g*. local yoghurt (Bongo), butter, (‘Ishabwe’) and consumption of locally produced milk products, were significantly associated with the occurrence of brucellosis among humans (*p* < 0.05) (Table 2).

### Multivariable analysis results

From Table 3, a final model was fitted to measure the relationship between *Brucella* seropositivity and independent variables. Both socio-demographic and potential risk factors that showed *p-*values <0.2 in the bivariate analysis were considered and included in the final multivariable regression model.

**Table 3:** Multivariable analysis of risk factors for occurrence of brucellosis among human participants

Variable	Adjusted OR	*p*-values	95 % CI
Residence			
Urban	1		
Rural	3.16	0.02	1.16, 8.56
Marital status			
Married	1		
Single	2.44	0.04	1.05, 5.68
Widowed	2.93	0.09	.85, 10.16
Divorced	0.45	0.32	0.09, 2.18
Exposure to animals at home			
No	1		
Yes	1.4	0.42	0.63, 3.12
Slaughter			
No	1		
Yes	1.46	0.55	0.42, 5.04
Processing Milk Products			
No	1		
Yes	1.26	0.60	0.53, 3.02
Consume locally processed milk products			
No	1		
Yes	2.54	0.03	1.12, 5.78
Assist animals giving births			
No	1		
Yes	1.56	0.45	0.50, 4.89
Drinking animal’s urine			
No	1		
Yes	1.11	0.91	0.19, 6.48
Sharing water points with animals			
No	1		
Yes	1.89	0.35	0.50, 7.11

Number of observations = 235; Hosmer and Lemeshow goodness-of-fit test, *p* = 0.23

Living in rural setting (OR 3.20, 95 % CI 1.17–8.78), consumption of locally processed milk products (OR 2.56, 95 % CI 1.12–5.85) and being single (OR 2.46, 95 % CI 1.05–5.74), increased the odds of being infected with *Brucella* among humans (Table 3).

## Discussion

Brucellosis occurs naturally in animals, while humans get infected through contact with the infected animal and consumption of contaminated animal products [2, 6, 7]. Therefore its prevalence in humans tends to correspond to that in animals [6, 40].

In the present study, human brucellosis seroprevalence was reported to be high (17 %) and this parallels with animal brucellosis prevalence that has been reported to range from 10.2 to 25.7 % in cattle [22]. Thus it is most probable that the animals in our study area are infected and serve as reservoirs and sources for the human brucellosis. The reported prevalence is higher compared to previous studies that reported prevalence of 10 % and 13.3 %, respectively among abattoir workers and individuals reporting to hospital with febrile illnesses in Uganda [12, 29]. This suggests that humans in pastoral communities who depend entirely on animals are at more risk of the disease [41]. This is because the participants in this study were from communities known to habitually consume raw milk and milk products, in addition to processing milk products, which are major risk factors for brucellosis in humans [1, 2, 41]. However, higher prevalence has been reported elsewhere; a 21.2 % prevalence was reported among patients with febrile clinical signs, 44 % among butcher workers in northern Nigeria [42, 43], while 40 % was reported in Libya [25]. In Libya, the prevalence was attributed to the culture and tradition of consuming raw milk and milk products by majority of pastoralists [44]. On the contrary, a study in Egypt reported a much lower prevalence of brucellosis in humans (8 %) following implementation of relevant control measures [45].

In this study, we identified three important risk factors for human brucellosis *i.e.* living in rural settings, consumption of traditionally prepared milk products and being single. Individuals who lived in the rural areas were three times more likely to be *Brucella* seropositive compared to their counter parts who lived in urban areas. This is in concordance with the studies done in Asian countries *e.g*. Iran [46], Palestine [47], Lebanon [48] and Saudi-Arabia [49] that reported high incidences of brucellosis among humans who stayed in rural areas. The underlying fact is that, people in rural areas tend to be more in close contact with animals which are known reservoirs for human brucellosis [6]. The observed 7.9 % seroprevalence among the urban people could be due to consumption of unpasteurized milk and milk products since boiling of milk is a common practice among consumers but unlike among pastoralists [50, 51].

Consumption of unpasteurized milk products was associated with the occurrence of brucellosis in line with various studies that reported similar findings [47, 52, 53]. This strengthens the argument that humans get infected through consumption of contaminated animal products as reported in other studies [44, 54]. Traditionally, the people in the study area consume raw milk and milk products. This is a common practice among males and the elderly who take milk directly from animals while they traditionally tend to them especially during field grazing. This could also explain why the *Brucella* seroprevalence was high among males compared to females and among elderly above 60 years though it was not statistically significant. This is in concordance with a study by Kalaajieh [48] who also reported no gender difference in occurrence of brucellosis although males contributed more cases. This is supported by other studies that reported more males than females as infected [25, 55, 56]. In contrast, other studies in Chad and Tanzania [57, 58] found age and sex to be associated with occurrence of brucellosis in humans.

Cases among males and the elderly being high are possibly because of traditional roles of these groups play in livestock care and management. Our results are in agreement with a study in Bangladesh where individuals of age group 40–80 years were more likely to be infected with brucellosis [56]. Another study in Lebanon reported that brucellosis cases increased with age group [48]. However, a study carried out in Turkey reported cases to be relatively high among younger population; noteworthy is that raising of livestock in Turkey begins at a younger age [59]. In the present study, the young population was school going and middle aged individuals involved in business. Cases of brucellosis in old groups could be explained by consumption of unpasteurized milk and milk products.

It is worth reporting that, during field grazing, some participants reported to drink cow’s urine as they believe it is pure and medicinal. It is reported that those who drunk animal’s urine, reported high cases (42.9 %) of brucellosis since urine is also a known fluid that risk to acquisition of brucellosis.

Other studies reported consumption of raw milk as risk factors for brucella infection [50, 59–61]. Indeed, we have demonstrated that individuals with a history of consumption of raw milk were more likely to be infected (20 % versus 16.6 %), though it was not statistically significant. A study in Bangladesh [56], also showed that brucellosis cases were high among those with the history of consuming raw milk (11.4 %) than those who did not have a history of consuming raw milk (3.9 %). This clearly explains that human brucellosis is contracted from animals reservoirs, milk being a major vehicle for the infection [1, 6, 50, 60, 61].

It was interesting to observe that being single was associated with the occurrence of brucellosis in humans. This could be explained by the fact that this group is the main consumer of milk and milk products despite the hygiene and safety of the product, since it is readily available in the informal markets as highly nutritious fast foods in this community. This is also in line with a study in Pakistan which demonstrated that married people have greater preference for packed milk compared to the unmarried [62]. In USA, the NPD’s *National Eating Trends*® research also reported young adults as main consumers of yogurt [63] because this was ready to eat food by this group of people who most likely had prior exposure of eating yogurt during breakfast.

There was no statistically significant correlation between occurrence of brucellosis and contact with home-owned animals, assisted animal parturitions, participating in milking and slaughter of animals, and sharing water points with animals. These findings are in contrast with a previous study that reported that brucellosis occurrence in humans was associated with contact with domestic animals [61], exposure to aborted animals and assisting animal parturition [52, 53] and or sharing of water sources with animals [28]. It should be noted that ours being a cross-sectional study, we largely depended on self-reporting by the participants who could have left out some possible factors associated to occurrence of brucellosis in humans as a big proportion had little or no knowledge of the disease. In this study, we used serological tests (SAT and RBT) characteristically of low specificity; they were used in combination to minimize measurement errors of false positives. Nevertheless, the high prevalence here reported points to a real problem institution of relevant control programs in the area.

## Conclusion

Brucella infection among the people living in agro-pastoral communities is an important public health problem in Uganda. This study revealed consumption of locally processed milk products, being single and living in rural area as risk factors associated with the brucella infection in humans.

Public awareness campaigns especially in rural agro-pastoral communities to disseminate knowledge about brucellosis and associated risk factors should be prioritized. Consumption of unpasteurized milk products should particularly be discouraged.

## Availability of data and materials

Not applicable.

## Abbreviations

SAT: Serum Agglutination Test
RBPT: Rose Bengal Plate Test
